# A Double-Blind Randomized Comparison Trial of Postoperative Pain in Patients Undergoing Total Shoulder Arthroplasty Who Receive Interscalene Blocks with near Equipotent Doses of Plain 0.5% Bupivacaine vs. Liposomal Bupivacaine

**DOI:** 10.3390/jcm15093434

**Published:** 2026-04-30

**Authors:** Johnny K. Lee, Rebecca Shamberg, Andrew R. Locke, Chi Wang, Steven Levin, Jason Koh, Laura Eldridge, Steven B. Greenberg

**Affiliations:** 1 Department of Anesthesiology, Critical Care & Pain Medicine, Endeavor Health, Evanston, IL 60201, USA; johnny.lee@endeavorhealth.org (J.K.L.);; 2 Department of Anesthesiology and Critical Care, Pritzker School of Medicine, University of Chicago, Chicago, IL 60637, USA; 3 Department of Statistics and Methodology, Endeavor Health, Evanston, IL 60201, USA; 4 Department of Orthopaedic Surgery, Endeavor Health, Evanston, IL 60201, USA; 5 Department of Orthopaedic Surgery & Rehabilitation Medicine, Pritzker School of Medicine, University of Chicago, Chicago, IL 60637, USA

**Keywords:** liposomal bupivacaine, total shoulder arthroplasty, interscalene block, regional anesthesia

## Abstract

**Background/Objectives**: Interscalene brachial plexus block (ISB) is a common regional anesthesia technique for analgesia in patients undergoing shoulder surgery. Liposomal bupivacaine (LB) was developed to prolong analgesia duration; however, the existing literature demonstrates mixed results regarding its efficacy. This study aimed to compare the analgesic effectiveness of near-equipotent doses of LB and plain bupivacaine (PB) for patients undergoing total shoulder arthroplasty (TSA). **Methods**: This prospective double-blinded randomized controlled trial enrolled 78 elective TSA patients. Participants were randomized to receive an ISB with either 36 mL of 0.5% PB (180 mg) or a mixture of 10 mL of LB, 20 mL of 0.25% PB, and 6 mL of saline (183 mg). The primary outcome was the proportion of patients with clinically tolerable pain scores (visual analog scale (VAS) ≤ 4) on postoperative day (POD) 1 in each group. Secondary outcomes included the proportion of patients with clinically tolerable pain scores on POD 2–5, overall pain scores in the post-anesthesia care unit (PACU) and on POD 1–5, Quality of Recovery Survey-15 (QoR-15) scores on POD 1–5, analgesic consumption on the day of surgery and on POD 1–5, and adverse events. **Results**: A total of 67 patients completed the study. There was a statistically significant increase in median body mass index (BMI) in the PB vs. LB group (30.0 (27.4–33.1) vs. 27.0 (24.3–29.4), *p* = 0.0197). All other demographic characteristics were comparable between groups. There was no difference in the primary outcome or any of the secondary outcomes. **Conclusions**: LB did not reduce postoperative pain compared to PB. Larger, multicenter studies are warranted to further evaluate the clinical benefit of LB in this population.

## 1. Introduction

Postoperative pain control remains a critical challenge in patients undergoing the increasing number of total shoulder arthroplasties and reverse total shoulder arthroplasties performed in the U.S. [1]. Effective analgesia is important not only for patient comfort but also to minimize opioid use, reduce hospital length of stay, and enhance overall recovery [2].

The interscalene brachial plexus block (ISB) is a common regional anesthesia technique used to provide optimal analgesia for shoulder surgery [3]. Traditional ISB commonly uses plain local anesthetics such as bupivacaine, which provides effective analgesia but is limited by its relatively short duration of action. Plain bupivacaine (PB) is often cited as having a duration of action less than 24 h, with several studies reporting 8–12 h [4,5]. This short duration of action is often insufficient to cover the most painful early postoperative period. In order to extend analgesia, the liposomal formulation of bupivacaine (liposomal bupivacaine, LB) was developed. The liposomal formulation prolongs release of the drug and possibly extends the analgesic effect for up to approximately 72 h after administration. LB was approved by the Food and Drug Administration for single-injection ISB in 2018 [6,7]. While the longer duration of action could theoretically provide an advantage in postoperative pain management, conflicting results have been found surrounding its efficacy. A recent randomized controlled trial found a statistically significant but not clinically meaningful reduction in opioid consumption over the first 72 h following total shoulder arthroscopy using LB over PB [8]. Several other articles showed no advantage to using LB over PB with regards to pain relief and decreasing opioid consumption [5,9,10].

Due to the uncertainty of clinical benefit, our research group performed a retrospective quality improvement study that demonstrated a statistically significant greater proportion of patients receiving LB in an ISB nerve block that had clinically tolerable pain scores (mean visual analog scale (VAS) ≤ 4) in the first 72 h, had a reduction in overall pain scores, and had a reduction in total opioid consumption [11]. These results prompted us to conduct the current prospective randomized controlled trial. The present study aimed to determine the clinical impact of supplementing PB with LB during ISB anesthesia for total shoulder arthroplasty (TSA). We hypothesize that the addition of LB will lead to a greater proportion of patients with clinically significant tolerable pain scores (VAS ≤ 4) on postoperative day (POD) 1.

## 2. Materials and Methods

This prospective randomized controlled trial enrolled elective surgery patients undergoing a primary anatomical total shoulder arthroplasty or reverse total shoulder arthroplasty within the Endeavor Health Orthopedic and Spine Institute between October 2023 and October 2025. The study was designed and reported according to Consolidated Standards of Reporting Trials (CONSORT) guidelines (Supplementary Materials). It was approved by the Endeavor Health IRB (IRB number EH23-069) and registered with clinicaltrials.gov under the identifier NCT05900427. All participants provided written informed consent prior to their participation in the study. Included subjects were male or female patients between 18 and 90 years old, weighing ≥60 kg at the time of surgery, and able to consent in English. Patients who had emergency orthopedic surgery cases, revision surgeries, multiple surgeries during one hospital stay, severe liver/kidney disease defined as a diagnosis of end-stage renal disease (ESRD) defined as being on dialysis, or pregnancy were excluded from enrollment. Additionally, any patients who recently received another local anesthetic block prior to the interscalene block, had an allergy or any contraindication to local anesthetics used in the study, or were contraindicated for use of liposomal bupivacaine were excluded.

Prior to surgery, all subjects were sent a Quality of Recovery-15 (QoR-15) survey electronically to document their baseline score. Survey data were collected and managed using REDCap 12.0 (Research Electronic Data Capture) electronic data capture tools hosted at Endeavor Health [12,13]. REDCap is a secure, web-based software platform designed to support data capture for research studies. Any subjects who did not complete the survey electronically had their responses recorded verbally by study personnel in the preoperative holding area prior to surgery.

A randomization allocation table was generated by the study biostatistician and used to create an electronic randomization module in REDCap that assigned each subject to receive an interscalene block in either the PB or LB group prior to surgery. Doses were equi-volume and nearly equipotent with the PB group consisting of 36 mL of 0.5% bupivacaine (180 mg) and the LB group of 10 mL of liposomal bupivacaine, 20 mL of 0.25% bupivacaine, and 6 mL of saline (183 mg).

### 2.1. Perioperative Management

On the day of surgery, those subjects enrolled in the study were randomized to a treatment group. All enrolled subjects received a single-injection interscalene block prior to surgery under ultrasound guidance and sedation with midazolam and up to 100 mcg of fentanyl at the anesthesia provider’s discretion. The interscalene blocks were performed by the attending anesthesiologist or by an anesthesiology resident under the supervision of an attending anesthesiologist. All interscalene blocks were performed in the preoperative holding area under ultrasound guidance per institutional practice prior to surgery.

The study drug was prepared by the attending anesthesiologist and overseen by the assigned research team member to confirm the dosage. Since it is a standard of care at our institution during regional anesthesia to aspirate prior to any injection to ensure that the needle is not intravascular, the anesthesiologist must be able to visualize the tubing and syringe prior to injection. Therefore, the tubing has to be transparent. Because liposomal bupivacaine is white in color and plain bupivacaine is clear, it would be impractical to blind the anesthesiologist in the study. All possible measures were taken to restrict the blinding to all personnel except the anesthesiologist and anesthesia research team member by limiting personnel in the room for the block procedure and documenting the administered block medication as “Study Drug” in the medical record. The anesthesia research team member was required to be unblinded, as they were responsible for using the randomization module in REDCap to assign the participant to the treatment group on the day of surgery, as well as to observe the block procedure to ensure it was performed as described in the protocol. All other individuals, including the surgical team, pre- and postoperative nursing staff, statisticians, and subject, were blinded. Concealment of allocation was ensured by using the electronic REDCap randomization module to randomize all study participants on the day of surgery once they checked in for their procedure. Using the REDCap module to generate the randomization ensured the next assignment remained unknown until the patient was enrolled.

All surgical procedures were performed by one of four orthopedic surgeons who routinely perform total shoulder arthroplasties at Endeavor Health. Once in the OR, induction drugs for general anesthesia were at the discretion of the anesthesia providers. Patients received approximately 2–3 mg/kg of propofol, 0.6 mg/kg of rocuronium or 1 mg/kg of succinylcholine, and 100 mcg of fentanyl. All subjects enrolled in the study received the standard general anesthetic with inhaled sevoflurane titrated to a Bispectral Index (Medtronic, Minneapolis, MN, USA) of 40–60 during surgery. Fentanyl was used for analgesia at the discretion of the anesthesia provider. Fentanyl was dosed in 50 mcg boluses based on a 20% increase in heart rate and/or blood pressure from baseline. Subjects were given rocuronium exclusively for maintenance of intraoperative neuromuscular blockade, titrated to a twitch count of 2–4/4 throughout the intraoperative case. All subjects were administered sugammadex or neostigmine and glycopyrrolate in anticipation of extubation. All patients received 4 mg of dexamethasone and 4 mg of ondansetron for postoperative nausea and vomiting. Following surgery, all patients were brought to the post-anesthesia care unit (PACU) where nurses assessed the subjects’ pain by using visual analog scales (VAS) (0–10). For pain scores ≥ 4, subjects received hydromorphone (0.2–0.4 mg) every 5 min as needed, which is the standard anesthesia PACU order at our institution.

After discharge from the PACU, all patients were either discharged home the same day from the Ambulatory Care Unit or admitted into the hospital with a planned stay overnight and most frequently discharged the next day. All patients had their pain managed by their respective surgical team following PACU discharge in accordance with the surgeon’s routine prescribing patterns. Depending on the pain and medications needed during the hospitalization, the standard outpatient pain medications were most often hydrocodone/acetaminophen (10/325 mg) or tramadol (50 mg).

### 2.2. Data Management and Outcomes

The primary outcome of this study was the proportion of patients in each cohort who reported clinically significant tolerable pain scores (VAS ≤ 4) on postoperative day (POD) 1. At our institution, patients who report a VAS > 4 are given opioid pain medications in the PACU per our study protocol. This is why any pain scores ≤ 4 were considered clinically tolerable. Published guidelines on administering VAS assessments to postsurgical patients confirm that scores ≤ 4 include none or mild pain, supporting our definition of clinically tolerable [14]. Multiple secondary outcomes were recorded and include: proportion of patients in each cohort with clinically tolerable pain scores in postoperative day (POD) 2–5, difference in VAS pain scores (0–10) in each group at PACU admission/discharge and POD 1–5, difference in Quality of Recovery Survey-15 (QoR-15), opioid use in morphine milligram equivalents (MME) the day of surgery and POD 1–5, non-opioid analgesic (NSAIDs, acetaminophen, gabapentanoids, skeletal muscle relaxants) use the day of surgery and POD 1–5; and adverse block events.

Study personnel collected patient information, including demographics and surgical data, from the EMR. The demographics data collected included age, sex, race, body mass index (BMI), medical history, surgical history, prior and current medications, alcohol and tobacco use, and ASA status. The surgical information collected included the type of surgery, length of surgery, hospital admission/outpatient surgery, hospital length of stay, surgical complications, and block complications. Additionally, patient VAS pain scores upon PACU admission, PACU discharge, and hospital discharge were recorded. Following surgery, study participants were sent electronic surveys using REDCap on postoperative days 1–5 where they were asked to record their VAS pain score, QoR-15, pain medications taken, and any adverse events. The adverse event survey screened for nerve block-related side effects such as ringing in the ears, metallic taste, seizures, irregular heartbeat, or persistent numbness, tingling, or weakness in the affected arm, as well as any hospital admissions or emergency department visits. Participants were also sent a final survey on postoperative day 30 to document any adverse events they had. Those participants who were unable to complete the surveys electronically were contacted by study personnel to provide responses via phone. Those patients who were admitted to the hospital following PACU discharge had all in-hospital analgesic consumption recorded by study staff from the EMR. Following data collection, all recorded dosages of both in-hospital and outpatient opioid pain medications were converted into morphine milligram equivalents (MMEs) using a standard conversion table prior to data analysis (Appendix A).

### 2.3. Statistical Analysis

Data was summarized using frequencies and the percentage for categorical data and mean and standard deviation (parametric) or median and interquartile range (IQR) (non-parametric) for numerical data. Categorical data between groups was compared using chi square test or Fisher exact test (cell frequency < 5) and continuous data was compared using *t*-test and Mann–Whitney U test depending on whether a mean or median was presented. Mixed-effects regression model accounting for missing data and correlation structure was employed to analyze longitudinal data, including VAS pain score, QoR 15, and oral MMEs. Multiplicity adjustment was performed using Bonferroni correction method. For the primary endpoint (proportion of patients with tolerable pain on postoperative day one), per-protocol analysis was used. Additionally, intention-to-treat analyses were performed as sensitivity analyses in which missing primary outcome data were handled using best-case (tolerable pain) and worst-case (intolerable pain) assumptions. For the secondary endpoints (VAS pain score, QoR 15, and oral MMEs), intent-to-treat analysis was employed. All data analyses were conducted using SAS 9.4 (SAS Inc., Cary, NC, USA). A *p* value less than 0.05 was considered statistically significant. Power analysis: we conducted power analysis using the baseline event rates of 69% (LB) and 44% (PB) from our pilot data with an estimated effect size of 25% absolute difference (OR = 2.83). To achieve a power of 80% with an alpha = 0.05, a total of 122 patients (*n* = 61 in each group) were needed. We proposed to enroll a targeted number of 128 patients to account for 5% attrition rate.

### 2.4. Protocol Deviation

This study was terminated early. After 61 patients had completed the study, which was half of the original sample size, an interim analysis was conducted. Results of this analysis showed no difference between the primary or secondary outcomes and determined that the effect size was much smaller than expected, and the target sample size was revised to 247 to maintain 80% power. Due to resource limitations and the lack of difference observed between study groups after the interim analysis, the study was closed prematurely. Six patients who had been enrolled during the interim analysis period completed enrollment, leading to a total of 67 patients who completed evaluation during the study.

## 3. Results

**Figure 1:**
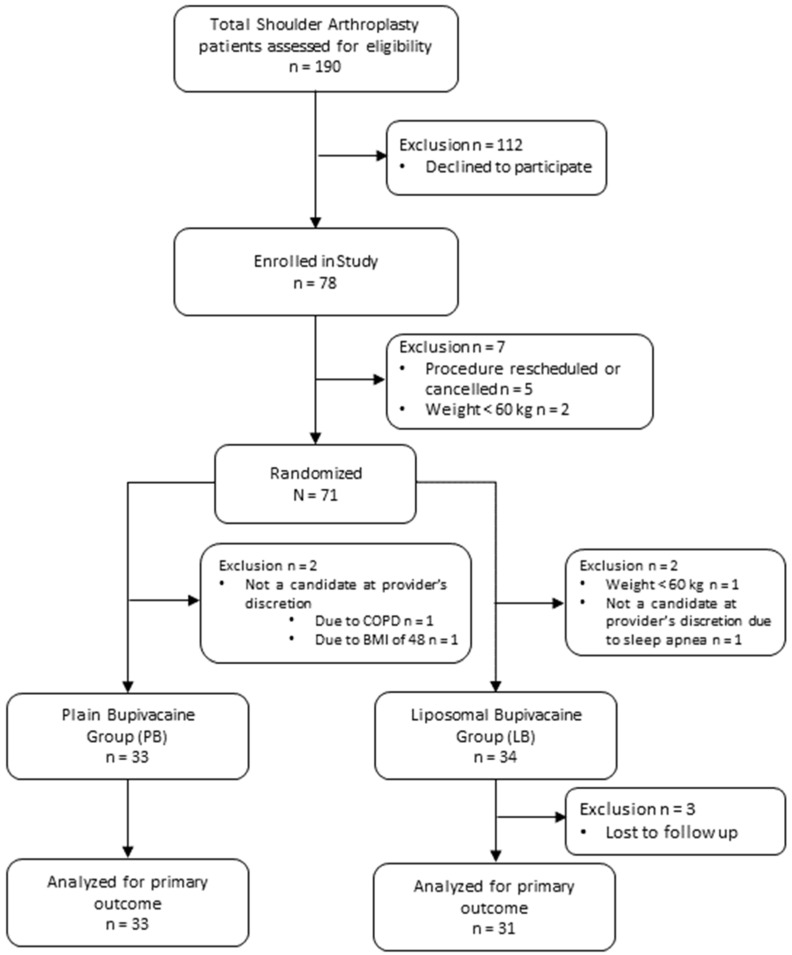
CONSORT flow diagram of randomization and exclusion of patients in the study.

A total of 78 patients were enrolled in the trial, and 67 patients completed the study based on the previously outlined criteria (Figure 1). Of the 67 patients who completed the study, 52 (77.6%) were reverse total shoulder arthroplasty and 15 (22.4%) were total shoulder arthroplasty, though procedure type was balanced between groups (Table 1). Subgroup analyses were not performed due to the small sample size of anatomic TSA and the comparable distribution of procedures between groups. Five patients were withdrawn due to rescheduled or cancelled procedures, three patients were withdrawn due to weighing <60 kg on the day of surgery, and three patients were withdrawn due to not being candidates for LB at the discretion of their anesthesia provider. Of these three patients, one patient had a COPD diagnosis, one patient had a sleep apnea diagnosis, and one patient had a BMI of 48, and for each case, the anesthesia attending was not comfortable with the patient receiving an interscalene block due to the increased safety risks.

Any patients who did not provide complete follow-up survey data for postoperative days one through five were excluded from analyses for those specific days but included in the analysis when complete data were available. Subsequently, the total N is less than 67 for VAS and QoR-15 variables across postoperative days one through five (See Table 2 and Table 3 for specific sample sizes at each time point). Overall, 4.5–12.1% of survey responses were missing on each study day. When stratified by study group, the same range of 4.5–12.0% of data was missing. Given the overall consistent level of missing data between groups on each day, the assumption of independence for the diagonal covariance is appropriate.

### 3.1. Demographics

There was a statistically significant increase in median BMI in the PB group (30.0 (27.4–33.1)) vs. the LB group (27.0 (24.3–29.4)) (*p* = 0.0197) (Table 1). There were no significant differences with regards to all other demographic characteristics between groups. Additionally, hospital LOS, procedure type, surgery type, intraoperative parameters, surgery type (outpatient vs. hospital admission), and hospital pain scores were not significantly different between groups (Table 1).

### 3.2. Primary/Secondary Outcomes

The absolute proportion of patients with clinically tolerable pain scores was 31.3% (20 out of 64)—18.8% (12 out of 64) for those who had taken liposomal bupivacaine and 12.5% (8 out of 64) who had taken plain (Table 2). Liposomal had an effect size of 1.4 [95% CI 0.8–2.3], and plain had an effect size of 0.7 [95% CI 0.4 to 1.3] (Table 3). There was no statistically significant difference among the two groups with respect to the proportion of patients with clinically tolerable pain scores on postoperative day one (*p* = 0.2121). Furthermore, there was no statistically significant difference in the proportion of patients with clinically tolerable pain scores on postoperative days two through five between the two treatment groups. In addition, average VAS pain scores from PACU through postoperative day 5 were not significantly different between the treatment groups (Table 4). In a mixed-effect model for VAS scores with type of bupivacaine and postoperative day, type of bupivacaine was not statistically significant (*p* = 0.2979), but there was a statistically significant difference in postoperative days (*p* ≤ 0.0001) (Table 5). The interaction between type of bupivacaine and day was not significant, so the day did not affect the significance of the bupivacaine type (*p* = 0.1671) (Table 5).

Using a per-protocol analysis, the difference in proportion of patients with tolerable pain on POD between LB and PB groups was 38.7% (12/31) vs. 24.2% (8/33) (*p* = 0.2121) (Table 2). Three patients in the LB group had missing data for the primary outcome. Sensitivity analyses were performed to assess the impact of this missing data. Using an intention-to-treat analysis with a worst-case assumption (all missing subjects had intolerable pain), the difference between LB and PB groups was 35.3% (12/34) vs. 24.2% (8/33) (*p* = 0.3229). Under a best-case assumption (all missing subjects had tolerable pain), the proportion of subjects with tolerable pain was 44.1% (15/34) vs. 24.2% (8/33) (*p* = 0.0867) for the LB and PB groups, respectively. In both scenarios, the results and conclusion of the study remain the same.

### 3.3. Quality of Recovery Survey-15 Scores

QoR-15 survey scores were not significantly different between the LB and PB groups at baseline or on postoperative days one through five (Table 6). Change-from-baseline analysis was not considered because baseline values were well balanced between groups, minimizing the likelihood of confounding and making between-group comparisons of absolute postoperative scores appropriate and sufficient. In a mixed-effect model for QoR-15 scores with type of bupivacaine and postoperative day, type of bupivacaine was not statistically significant (*p* = 0.8763) nor was there a statistically significant difference when looking at postoperative day (*p* = 0.3078) (Table 5). There was also no statistical difference when interaction between bupivacaine type and day was examined (*p* = 0.8455) (Table 5).

### 3.4. Opioid Consumption in MME

MME consumption from PACU through postoperative day 5 was also not found to be significantly different in the LB group when compared to PB (Table 6). In a mixed-effect model of MME consumption with type of bupivacaine and postoperative day, type of bupivacaine was not statistically significant (*p =* 0.5686), but there was a statistically significant difference depending on the day (*p* = 0.0039), indicating there is a difference in MME consumption depending on the day (Table 5). There was no statistical difference when interaction between bupivacaine type and day was examined (*p* = 0.2434) (Table 5).

### 3.5. Non-Opioid Analgesic Consumption

Acetaminophen and ibuprofen consumption was not found to be significantly different between groups from PACU through postoperative day 5.

### 3.6. Safety

Eighteen patients in both the PB and LB groups reported procedure-related events that are summarized in Table 7. All events were known potential interscalene block complications and followed expected frequencies. One serious event was reported in the LB group that was not related to the study drug or procedure.

## 4. Discussion

This double-blinded randomized controlled trial suggested no difference in the proportion of patients with clinically tolerable pain at post operative day 1 or at any point after a total shoulder arthroplasty surgery when performing an interscalene block with LB combined with plain bupivacaine compared to PB alone despite nearly equipotent doses. Although we report near-equipotent milligram dosage, according to the manufacturer, one 10 mL vial contains 133 mg of LB, which is equivalent to 150 mg of bupivacaine HCl [15]. Thus, the LB group had a slightly higher amount of bupivacaine (200 mg) than the PB group (180 mg). Even with the slightly higher amount of bupivacaine in the LB group, there still were no differences between groups, suggesting a lack of efficacy of enhanced analgesia of LB vs. PB. Additionally, analysis of our secondary outcomes did not show a statistically significant difference in mean VAS scores, quality of recovery scores, or opioid consumption between the groups.

The differences in findings with respect to the present trial when compared to the authors’ retrospective study may be explained by several points. The retrospective study did not solely compare interscalene blocks with the addition of LB to those with PB alone. In fact, the non-liposomal bupivacaine group included ropivacaine and mepivacaine, and they were not delivered to patients in equipotent doses. The heterogeneity in dosing and type of local anesthetic in the non-LB group may have accounted for the greater observed difference in the retrospective study. In addition, in the time since the previous quality improvement retrospective study, our orthopedic services were consolidated to one large orthopedic and spine specialty hospital. At this site, anesthesia care is consistently provided by experienced regional anesthesiologists with high proficiency in regional anesthesia techniques and medications. The reduced variability in provider expertise and comfort with regional anesthesia techniques and medications may have resulted in some of the observed outcomes.

Other reasons may have contributed to lack of perceived differences. While the manufacturer reports release of bupivacaine up to 72 h, previous studies have reported a bimodal pharmacokinetic plasma concentration profile, with an initial peak of free bupivacaine followed by a second, albeit lower, peak from sustained release from the liposomes [16]. It is possible that this second peak fails to reach minimum effective concentration to provide adequate pain relief. Additionally, the concomitant use of effective multimodal analgesia may have created a ceiling effect and obscured small differences between the LB and PB groups by significantly reducing pain scores and opioid consumption across all groups [17]. While withholding multimodal therapy might have produced different results, such an approach would have been ethically unsound and would not reflect the authors’ current clinical practice.

The present study has several strengths. Our study was performed at a large orthopedic and spine specialty hospital with anesthesia teams supervised by proficient regional anesthesiologists. The teams consisted of either regional attending anesthesiologists or regional anesthesiologists supervising anesthesia residents, CRNAs, or SRNAs. The diversity of the anesthesia team means that the results of this study could be applicable to academic or community hospitals. Additionally, the use of near equipotent dosing in the present study to compare standard PB and LB distinguishes our study from the current literature.

While LB is theorized to reduce pain and opioid consumption following shoulder surgery compared to PB, the literature suggests mixed results. Elmer et al. reported that PB did not demonstrate non-inferiority when compared to a combination of bupivacaine and LB with regard to 72 h opioid consumption; however, this difference was clinically insignificant [18]. Their control group also only received 100 mg of bupivacaine compared to 183 mg in the liposomal group. Virk et al. also compared liposomal and plain bupivacaine for ISB in 150 total shoulder arthroplasty patients and found that opioid consumption was reduced in the PB group by 24 MME from 24 to 72 h after surgery, though this equates to less than 100 mg of tramadol and was therefore deemed not clinically significant [8]. They also found a statistically, but not clinically significant, difference in pain intensity on postoperative days 7 and 14. However, this study also did not use near equal doses of bupivacaine; their LB group received the same regional anesthetic protocol as the present study, yet their control group received only 20 mL of 0.5% bupivacaine compared to 36 mL in the present study. Similarly, in a trial of 104 patients, Hattrup et al. found that ISB with 5 mL of 0.5% PB vs. 133 mg of LB with 7.5 mL each of 0.5% and 0.25% PB did not lead to any clinically significant difference in VAS pain scores, total MME consumption, or patient satisfaction with pain management [5]. In contrast, Finkel et al. reported that patients receiving ISB with 10 mL of 0.5% bupivacaine and 133 mg of LB had significantly lower pain scores at 24–48, 48–72, and 72–96 h after surgery compared to ISB with 20 mL of 0.5% bupivacaine with 5 mg of preservative-free dexamethasone plus 5 μg of epinephrine [19]. Once again, in contrast to the present study, this study did not use equipotent doses.

Our study has several limitations. First, this study was conducted at a single center and may not reflect practices performed elsewhere. Second, we originally powered the study to detect a 25% difference in the proportion of patients with clinical tolerable pain between the LB and PB groups. We determined that a total of 122 (61 in each group) patients were needed to achieve 80% power. However, after 61 patients had completed the study, we conducted an interim analysis and determined that the effect size was much smaller (38.5% vs. 26.7%, OR = 1.72) than expected. We would need to increase the sample size to 247 per group to maintain 80% power, making it logistically impossible to continue the trial to its planned completion, and the study was concluded early. Given that only 67 of the planned 122 participants completed the study, the trial may have been underpowered to detect clinically meaningful differences, which should be heavily considered when interpreting the negative findings. Third, a majority of our data collection relied on self-reporting after discharge from the hospital. This could result in inaccuracies compared to data collection in an inpatient setting, where research staff could have directly followed up with every patient. Nevertheless, the research staff did try and reach out to all patients who did not complete their REDCap surveys. However, the staff could not confirm the accuracy of the ones completed. It would also have been unethical to keep the patients admitted for the purposes of accurate data collection. Fourth, it is important to note that there was a statistically significant difference in the median BMI between the two groups (LB 27 vs. PB 30). A larger BMI associated with increased adipose tissue could create technical difficulties for an ultrasound-guided regional block such as poor image quality and needle visualization. Additionally, a larger BMI could also increase the patient’s volume of distribution, resulting in a need for higher analgesic doses for a similar effect. Considering these factors combined, one would expect worse pain outcomes. In our study, this would possibly result in the LB group having an advantage. However, post hoc multivariable analysis for pain tolerability controlling for BMI on POD 1 through POD 5 found that BMI was not a significant predictor for the primary or secondary outcomes. Additionally, given that there was no difference seen in the proportion of patients with tolerable pain, mean VAS scores, or opioid consumption, it is unlikely that this was clinically significant. Fifth, it is plausible that the PB block is initially denser in the first 12 h given that the amount of free bupivacaine is likely higher in the PB group compared to the LB group. Furthermore, intraoperative opioid consumption between groups was not recorded. It is possible that one study group received higher doses of opioid pain medications during surgery, with the potential to impact postoperative VAS pain scores. However, it is worth noting that the mean PACU opioid consumption for both groups was 0, which implies equal efficacy in the immediate perioperative period. Sixth, as shown in Figure 1, two participants from each study group were withdrawn from the study after randomization but prior to any study procedures taking place. This occurred because the assigned research staff member completed the randomization module in REDCap prior to the anesthesia attending physician deciding that ISB was not appropriate for these patients due to safety concerns. This may have introduced selection or allocation bias, as the decision to withdraw treatment was made after randomization had occurred and based on perceived clinical risk. Since two patients were withdrawn from each group, the overall balance between groups was likely unchanged. However, the authors acknowledge that these withdrawals could have weakened the internal validity of the study, and our findings should be interpreted with this in mind. Finally, four surgeons were involved in study procedures. However, the proportion of patients with clinically tolerable pain scores, overall VAS scores, and opioid consumption in MMEs were not significantly different when stratified by surgeon. Thus, the lack of any significant differences observed in the primary and secondary outcomes cannot be attributed to surgeon-specific variability.

Our study does suggest that when PB is administered, it should be done at optimized, near equipotent doses within a multimodal analgesic framework, as LB pain scores, quality of recovery, and opioid-sparing effects over the first 96 h are similar. These results may add to the current literature regarding the benefits and liabilities of using PB vs. LB.

## 5. Conclusions

In summary, this randomized study involving ISB performed with LB vs. PB with near equipotent doses of bupivacaine did not suggest a difference in the proportion of clinically tolerable pain or quality of recovery scores. However, due to terminating our study early, the study may have been underpowered to detect a difference. Further multicenter trials may be needed to confirm the present findings and to investigate other long-term outcomes in these patients that may be important in this patient population.

## Figures and Tables

**Table 1:** Patient demographics by study group.

Demographic Variable	Total	LB	PB	*p*-Value
N (%)	67	34 (51)	33 (49)	
Age				
Age [mean (SD)]	70 (8)	70 (9)	71 (7)	0.6533
Age [median (Q1–Q3)]	70 (66–75)	68 (66–75)	72 (66–75)	0.7345
Sex N (%)				0.3889
Female	32 (47.8)	18 (52.9)	14 (42.4)	
Male	35 (55.6)	16 (47.1)	19 (57.6)	
Race N (%)				0.3559
Caucasian	62 (88.6)	30 (83.3)	32 (94.1)	
Other/unknown	5 (7.5)	4 (11.8)	1 (3.0)	
ASA Status N (%)				0.5341
2	31 (46.3)	17 (50.0)	14 (42.4)	
3	36 (53.7)	17 (50.0)	19 (57.6)	
Physical Measurements				
Height [mean (SD)]	1.7 (0.1)	1.7 (0.1)	1.7 (0.1)	0.9269
Height [median (Q1–Q3)]	1.7 (1.6–1.8)	1.7 (1.6–1.8)	1.7 (1.6–1.8)	0.9499
Weight [mean (SD)]	84.5 (16.2)	81.2 (14.2)	87.8 (17.7)	0.0982
Weight [median (Q1–Q3)]	81.2 (72.6–95.3)	79.1 (72.6–89.8)	83.9 (73.5–99.8)	0.1657
BMI [mean (SD)]	29.2 (5.1)	28.3 (5.6)	30.3 (4.3)	0.0928
BMI [median (Q1–Q3)]	28.3 (26.0–32.1)	27.0 (24.3–29.4)	30.0 (27.4–33.1)	0.0197
BMI of female subjects [mean (SD)]	30.4 (6.1)	29.5 (6.8)	31.6 (4.9)	0.3452
Alcohol/Tobacco Use N (%)				0.8112
Alcohol	15 (21.4)	7 (19.4)	8 (23.5)	
Cannabis	1 (1.4)	1 (2.8)	0 (0.0)	
Tobacco	20 (28.6)	9 (25.0)	11 (32.4)	
None	31 (46.3)	17 (50.0)	14 (42.4)	
System Comorbidities N (%)				
Liver	1 (1.5)	0 (0.0)	1 (3.0)	0.4925
Kidney	4 (6.0)	3 (8.8)	1 (3.0)	0.6135
Endocrine	15 (23.1)	5 (15.6)	10 (30.3)	0.1603
Respiratory	30 (44.8)	14 (41.2)	16 (48.5)	0.5475
Neurological disease	3 (4.6)	3 (8.8)	0 (0.0)	0.2399
Medical History N (%)				
Taking prescription medications	65 (97.0)	33 (91.7)	32 (94.1)	0.9829
History of prior surgery	64 (95.5)	33 (91.7)	31 (91.2)	0.5371
Procedure Type N (%)				0.4158
Reverse total shoulder arthroplasty	52 (77.6)	25 (73.5)	27 (81.8)	
Total shoulder arthroplasty	15 (22.4)	9 (26.5)	6 (18.2)	
Intraoperative Parameters				
Procedure duration [mean (SD)]	1.6 (0.5)	1.6 (0.5)	1.6 (0.5)	0.8145
Procedure duration [median (Q1–Q3)]	1.5 (1.3–2.0)	1.5 (1.3–2.0)	1.4 (1.3–1.9)	0.9151
Surgery duration [mean (SD)]	2.7 (0.7)	2.7 (0.7)	2.7 (0.7)	0.8817
Surgery duration [median (Q1–Q3)]	2.5 (2.2–3.1)	2.5 (2.2–3.1)	2.5 (2.3–3.0)	0.8263
Complications	2 (3.0)	0 (0.0)	2 (6.1)	0.2388
Surgery Type N (%)				0.6900
Hospital admission	26 (38.8)	14 (41.2)	12 (36.4)	
Outpatient	41 (61.2)	20 (58.8)	21 (63.6)	
Hospital Pain Scores				
Last hospital VAS [mean (SD)]	2.0 (2.2)	1.8 (2.0)	2.2 (2.4)	0.4744
Last hospital VAS [median (Q1–Q3)]	2 (0–3)	1 (0–3)	2 (0–3)	0.6643
Hospital LOS				
Hospital LOS in hours [mean (SD)]	18.6 (12.5)	18.0 (11.6)	19.2 (13.6)	0.7022
Hospital LOS in hours [median (Q1–Q3)]	9.8 (8.5–30.1)	9.8 (8.4–31.2)	10.5 (8.9–28.9)	0.9002

**Table 2:** Absolute proportions of pain tolerability on POD 1.

Tolerability	*N* = 64	*N* = 31	*N* = 33	*p*-Value
				0.2121
Tolerable (VAS ≤ 4)	20 (31.3)	12 (18.8)	8 (12.5)	
Intolerable (VAS > 4)	44 (68.8)	19 (29.7)	25 (39.1)	

**Table 3:** Effect size of pain tolerability on POD 1.

Statistic	Value	95% Confidence Limits	
Odds ratio	1.9737	0.6735	5.7843
Relative risk (liposomal bupivacaine)	1.3895	0.8488	2.2746
Relative risk (plain bupivacaine)	0.704	0.3882	1.2768

**Table 4:** Pain tolerability and VAS pain scores by study group.

VAS Variable	Total	LB	PB	*p*-Value
*N* (%)	67	34 (51)	33 (49)	
Proportion of Patients with Tolerable Pain *N* (%)				
PACU admission				0.9754
Tolerable (VAS ≤ 4)	63 (94.0)	32 (94.1)	31 (93.9)	
Intolerable (VAS > 4)	4 (6.0)	2 (5.9)	2 (6.1)	
PACU discharge				0.4275
Tolerable (VAS ≤ 4)	61 (91.0)	32 (94.1)	29 (87.9)	
Intolerable (VAS > 4)	6 (9.0)	2 (5.9)	4 (12.1)	
Day 1				0.2121
	*N* = 64	*N* = 31	*N* = 33	
Tolerable (VAS ≤ 4)	20 (31.3)	12 (38.7)	8 (24.2)	
Intolerable (VAS > 4)	44 (68.8)	19 (61.3)	25 (75.8)	
Day 2				0.4245
	*N* = 64	*N* = 32	*N* = 32	
Tolerable (VAS ≤ 4)	21 (32.8)	12 (37.5)	9 (28.1)	
Intolerable (VAS > 4)	43 (67.2)	20 (62.5)	23 (71.9)	
Day 3				0.2041
	*N* = 62	*N* = 31	*N* = 31	
Tolerable (VAS ≤ 4)	31 (50.0)	13 (41.9)	18 (58.1)	
Intolerable (VAS > 4)	31 (50.0)	18 (58.1)	13 (41.9)	
Day 4				0.3135
	*N* = 64	*N* = 32	*N* = 32	
Tolerable (VAS ≤ 4)	36 (56.3)	16 (50.0)	20 (62.5)	
Intolerable (VAS > 4)	28 (43.8)	16 (50.0)	12 (37.5)	
Day 5				0.1353
	*N* = 63	*N* = 32	*N* = 31	
Tolerable (VAS ≤ 4)	41 (65.1)	18 (56.3)	23 (74.2)	
Intolerable (VAS > 4)	22 (34.9)	14 (43.8)	8 (25.8)	
VAS Pain Score [Median (Q1–Q3)]				
PACU admission	0 (0–0)	0 (0–0)	0 (0–0)	0.7782
PACU discharge	0 (0–2)	0 (0–2)	0 (0–2)	0.7443
Day 1	5.4 (3.4–7.1)	5 (3.2–6.9)	6.4 (4.1–7.4)	0.1897
Day 2	5 (3.7–6.7)	5.1 (3.7–7.1)	5 (3.6–6.6)	0.7016
Day 3	4.1 (2.8–5.5)	4.7 (2.5–6.7)	4 (2.9–5.0)	0.2568
Day 4	3.8 (1.8–5.6)	4.1 (1.9–6)	3.3 (1.8–5)	0.4048
Day 5	3.2 (1.4–5)	3.7 (1.3–5.1)	3 (1.4–4.1)	0.6697

**Table 5:** Mixed-effects models for primary and secondary outcomes.

Parameter	Estimate	Confidence Intervals	*p*-Value
Pain Scores			
Intercept	6.2	5.4–7.0	<0.0001
Group (LB vs. PB)	0.1	−7.7	0.2979
Time (day)	−0.6	−0.5	<0.0001
Group × time	0.2	−0.7	0.1671
Oral MME Consumption			
Intercept	26.5	20.8–32.1	<0.0001
Group (LB vs. PB)	2.8	−15.1	0.5686
Time (day)	−2.8	−3.7	0.0039
Group × time	1.6	−5.3	0.2434
Total QOR Score			
Intercept	110.9	105.7–116.0	<0.0001
Group (LB vs. PB)	−0.6	−14.4	0.8763
Time (day)	0.9	−3.4	0.3078
Group × time	0.2	−4.7	0.8455

**Table 6:** QoR-15 scores and oral MME consumption by study group.

Score Variable	Total	LB	PB	*p*-Value
Median (Q1–Q3)	67	34	33	
Total QOR Score [Mean (SD)]				
	*N* = 60	*N* = 31	*N* = 29	
Baseline	122.3 (16.8)	122.3 (18.8)	122.3 (14.7)	0.9901
	*N* = 63	*N* = 32	*N* = 31	
Day 1	100.1 (21.9)	100.5 (21.5)	99.6 (22.6)	0.8739
	*N* =58	*N* = 29	*N* = 29	
Day 2	108.9 (18.4)	107.4 (18.7)	110.4 (18.3)	0.5480
	*N* =58	*N* = 29	*N* = 29	
Day 3	113.3 (16.6)	112.8 (15.4)	113.8 (18.1)	0.8274
	*N* = 59	*N* = 29	*N* = 30	
Day 4	115.8 (19.3)	116.2 (18.9)	115.5 (20.1)	0.8900
	*N* = 61	*N* = 31	*N* = 30	
Day 5	118.7 (16.5)	119.5 (16.0)	117.7 (17.2)	0.6706
Oral MMEs [Mean (SD)]				
PACU	0 (0–11.5)	0 (0–10)	0 (0–17.5)	0.4534
Day 1	37.5 (15–50)	38.8 (15–45)	30 (15–57.5)	0.6684
Day 2	25 (10–40)	25 (10–35)	20 (10–40)	0.8353
Day 3	15 (0–30)	20 (0–35)	15 (0–30)	0.5919
Day 4	5 (0–30)	12.5 (0–22.5)	0 (0–30)	0.6223
Day 5	0 (0–20)	12.5 (0–20)	0 (0–15)	0.1852
Total	110 (55–165)	105 (50–180)	111.3 (60–150)	0.9650 0.3452

**Table 7:** Safety endpoints by study group.

Type of Adverse Event N (%)	LB	PB
Persistent numbness, tingling, and weakness	5 (14.7)	7 (21)
Tinnitus	4 (11.8)	4 (12.1)
Metallic taste	3 (8.8)	1 (3.0)
ER or hospital admission lasting <24 h within 30 days of surgery	6 (17.6)	6 (18.2)

## Data Availability

The datasets used and/or analyzed in this study are available from the corresponding author upon reasonable request.

## Supplementary Materials

The following supporting information can be downloaded at: https://www.mdpi.com/article/10.3390/jcm15093434/s1, CONSORT 2025 checklist [20].

## Abbreviations

The following abbreviations are used in this manuscript:

ISB	Interscalene block
PB	Plain bupivacaine
LB	Liposomal bupivacaine
POD	Postoperative day
PACU	Post-anesthesia care unit
QoR-15	Quality of Recovery Survey-15
VAS	Visual analog scale
MME	Morphine milligram equivalent

## Appendix A

**Table A1:** Standard opioid-to-MME conversion chart.

Medication	Parenteral Dose (mg)	Oral Dose (mg)
Morphine (Roxanol, MSIR)	10	30
Hydromorphone (Dilaudid, Hydrostat)	1.5	7.5
Fentanyl (Duragesic)	0.1	--
Oxycodone (Roxicodone, Percocet)	--	20
Methadone (Dolophine)	Refer to Lexicomp Consider expert consultation	Refer to Lexicomp Consider expert consultation
Hydrocodone (Vicodin, Lortab, Norco)	--	30
Oxymorphone (Opana)	--	10 *
Tapentadol	--	100–150 *
Codeine	--	200
Meperidine (Demerol)	75	Not recommended
Buprenorphine (Buprenex)	0.4	--

* Estimated conversion factor which is not supported by published evidence.
